# Indocyanine green fluorescence imaging versus modified inflation-deflation method for intersegmental plane identification in thoracoscopic segmentectomy: a systematic review and meta-analysis

**DOI:** 10.1097/MS9.0000000000005444

**Published:** 2026-10-21

**Authors:** Ahmed Asif, Maheen Tariq, Muhammad K. Kirmani, Taimoor Qadeer, Muhammad Shahzaib, Zainab Ehsan, Ghina Fatima, Aryan Tareen, Anoud Khan, Hadiya Nadeem, Muhammad K. Buhadur Ali, Usama Jamil

**Affiliations:** a Department of Medicine and Surgery, King Edward Medical University, Lahore, Pakistan; b Department of Radiology, Feinberg School of Medicine, Northwestern University, Chicago, IL, USA; c Department of Medicine and Surgery, Shaikh Khalifa Bin Zayed Al Nahyan Medical and Dental College, Lahore, Pakistan; d Department of Medicine and Surgery, KRL Hospital, Islamabad, Pakistan; e Department of Medicine and Surgery, Lahore Medical and Dental College, Lahore, Pakistan; f Department of Medicine and Surgery, FMH College of Medicine and Dentistry, Lahore, Pakistan; g Department of Medicine and Surgery, Gomal Medical College, DI Khan, Pakistan; h Department of Medicine and Surgery, Ziauddin Medical College, Karachi, Pakistan; i Department of Medicine and Surgery, CMH Lahore Medical College, Lahore, Pakistan; j Department of Medicine and Surgery, Kabul University, Kabul, Afghanistan

## Abstract

**Background:**

Accurate identification of the intersegmental plane is essential to ensure both surgical safety and adequate oncologic margins during thoracoscopic segmentectomy. The modified inflation–deflation method (IDM) is commonly used but may be unreliable in patients with impaired pulmonary function. Indocyanine green fluorescence imaging (ICG-FI) has emerged as an alternative technique offering real-time perfusion-based visualization. This systematic review and meta-analysis compares perioperative and postoperative outcomes between ICG-FI and IDM during thoracoscopic segmentectomy.

**Methods:**

A systematic search of PubMed, Embase, Cochrane Library, and ScienceDirect was conducted up to August 2025, following PRISMA guidelines. Studies comparing ICG-FI with IDM for intersegmental plane identification in thoracoscopic segmentectomy were included. Primary outcomes included operative time, blood loss, intersegmental plane visualization time, and postoperative complications. Pooled effect sizes were calculated using fixed- and random-effects models, depending on heterogeneity.

**Results:**

Twelve studies comprising 1807 patients were included. Compared with IDM, ICG-FI significantly reduced operative time [mean difference (MD) −21.09 minutes, 95% CI: −33.24 to −8.95, *P* = 0.0007, I2 = 96%] and intraoperative blood loss (MD −12.29 mL, 95% CI: −21.21 to −3.37, *P* = 0.007, *I*^2^ = 81%). ICG also significantly shortened intersegmental plane visualization time (MD = –20.21 minutes; 95% CI: −26.49 to −13.92, *P* < 0.00001). Despite high heterogeneity (*I*^2^ = 100%), all included studies demonstrated a substantial and consistent advantage for ICG in delineating intersegmental planes. There was no significant difference in lymph node harvest, indicating preserved oncologic completeness. Most postoperative complications were comparable between groups; however, sensitivity analysis demonstrated a significant reduction in postoperative air leaks with ICG-FI. Length of hospital stay was also modestly shorter in the ICG-FI group.

**Conclusion:**

ICG-FI improves intraoperative efficiency and precision in thoracoscopic segmentectomy without compromising safety or oncologic adequacy. While postoperative complication rates are largely similar, ICG-FI may reduce air leaks and hospitalization duration. Further randomized trials are needed to confirm long-term outcomes and standardize fluorescence protocols.

## Introduction

Lung cancer has become the malignancy with the highest morbidity and mortality worldwide, making it a serious life-threatening disease^[^1^]^.

Traditionally, the management of patients with non-small cell lung cancer includes lobectomy with complete lymph-node dissection through a conventional thoracotomy or a thoracoscopic approach. However, with the advent of new technology and superior diagnostic techniques, an increasing number of lung cancer cases are being detected at earlier stages, with lesions commonly found to be less than 2 cm in size. These advancements have renewed interest in sublobar resections, segmentectomies in particular, as a preferred approach to resectable lesions. For lesions of this size, extensive research has provided conclusive evidence that anatomic segmental resection and pulmonary lobectomy have similar effects on patient outcomes^[^2,3^]^.

HIGHLIGHT

- Indocyanine green (ICG) fluorescence enables real-time perfusion mapping for precise intersegmental plane identification.
- A meta-analysis confirms that ICG fluorescence imaging (ICG-FI) significantly reduces operative time, blood loss, and visualization latency.
- Sensitivity analysis demonstrates a significant reduction in postoperative air leaks with the use of ICG-FI.
- ICG guidance shortens hospital stay while maintaining oncologic adequacy and lymph node yield.
- Fluorescence imaging offers a superior and efficient alternative to the modified inflation-deflation method.

Naturally, anatomic pulmonary segmentectomy has gained increasing prominence as a parenchyma-sparing surgical option for early-stage lung cancer and select benign pulmonary disease, especially in patients with reduced pulmonary reserve. While previously the standard of care was lobectomy, recent randomized data (such as the JCOG 0802/WJOG4607L trial) demonstrate that segmentectomy for small-sized (≤2 cm) semi-solid/solid nodules offers non-inferior survival and better preservation of lung function^[^4^]^.

A cornerstone of an effective segmentectomy is accurately identifying the intersegmental plane and achieving an adequate surgical margin. A variety of techniques have been developed to ensure adequate margins. The modified inflation–deflation method (IDM) remains widely practiced due to its simple approach. However, there are limitations in patients with reduced pulmonary reserve, COPD, and distorted anatomy. IDM can be technically unstable and challenging, especially in video-assisted thoracic surgery, because the lung may fail to deflate sufficiently, potentially obstructing the surgical view and affecting operative time^[^5,6^]^.

Near-infrared fluorescence imaging with systemic injection of indocyanine green (ICG) has emerged in recent years as an alternative. Misaki *et al* were among the first to test this approach by identifying the dominant pulmonary artery supplying the target segment using CT images. The artery was ligated, and ICG, which fluoresces under infrared light, was administered intravenously, enabling the team to mark the white-to-blue transitional zone with electrocautery and perform segmentectomy. This novel method allowed real-time visualization of perfused versus non-perfused lung, helping to achieve precise demarcation^[^4,6,7^]^.

Previous research and meta-analyses comparing these two methods recommend the regular application of ICG fluorescence to enhance the quality of segmentectomy and improve short-term outcomes^[^8^]^.

Despite promising results, comparative data are still inconclusive, and there is no clear consensus on which method yields superior margin success or long-term outcomes. This prompted us to conduct a systematic review and meta-analysis comparing intraoperative and long-term outcomes between the conventional modified IDM and ICG fluorescence imaging (ICG-FI) to identify intersegmental margins and achieve margin success in thoracic surgery. This study further builds on prior work by adding metrics of surgical precision and postoperative outcomes to expand existing knowledge regarding this novel technique in thoracoscopic segmentectomy.

## Methods

### Search strategy and selection criteria

We searched PubMed, Embase, the Cochrane Library, and ScienceDirect up to August 2025. We used a combination of MeSH terms and entry terms to conduct a comprehensive search. The main search terms used were “indocyanine green,” “segmentectomy,” and “thoracic surgery.” The detailed search strategy is provided in Supplemental Digital Content File S1, available at: https://links.lww.com/MS9/B410. This analysis was conducted according to the PRISMA guidelines. We registered this meta-analysis in PROSPERO (CRD420251133611).

### Inclusion and exclusion criteria

Studies with the following features were included: (1) the interventional group underwent thoracoscopic segmentectomy using ICG-FI to determine the intersegmental boundary line (IBL), without limitations regarding pathological classification, age, or gender; (2) the comparison group used the modified IDM to determine the IBL; (3) outcome measures included intraoperative indicators such as surgical time, intraoperative blood loss, and the time to presentation of the IBL, and postoperative indicators including complications, duration of chest tube drainage, and length of hospital stay; and (4) data preservation was complete and comprehensive. The exclusion criteria were as follows: (1) studies for which the full text could not be retrieved; (2) studies with incomplete or incoherent data reporting; (3) publication types such as reviews, case reports, and letters to the editor; (4) studies that did not report relevant outcome indicators; and (5) studies utilizing ICG for nodule localization only.

### Data extraction

Data were extracted by four independent researchers and included the first author’s name, publication date, country, surgical methods, operation time, intraoperative blood loss, number of complications, length of hospitalization, and postoperative complications.

### Quality assessment

The methodological quality of non-randomized studies was evaluated using the Risk of Bias in Non-randomized Studies of Interventions (ROBINS-I) tool, which evaluates bias across seven domains and categorizes the overall risk as low, moderate, serious, or critical. For randomized clinical trials, the Revised Cochrane Risk of Bias tool (RoB 2.0) was used to evaluate methodological quality; this tool helped us assess potential bias across key domains such as the randomization process, deviations from intended interventions, missing outcome data, accuracy of outcome measurements, and selective outcome reporting. Disagreements in the quality assessment were resolved through discussion. The results of the quality assessment tools were visualized using the robvis visualization tool. The AMSTAR 2 checklist used for methodological appraisal is provided in Supplemental Digital Content File S2, available at: https://links.lww.com/MS9/B411.

### Statistical analysis

Statistical analyses were conducted using Review Manager (RevMan) version 5.4. Pooled effect estimates were calculated as odds ratios (ORs) or risk ratios (RRs) for categorical outcomes, and mean differences (MDs) and standard deviations for continuous outcomes, each accompanied by 95% confidence intervals (CIs). Statistical heterogeneity across studies was assessed using the *I*^2^ statistic, with thresholds interpreted as follows: low heterogeneity (*I*^2^ < 50%) warranted the use of a fixed-effects model, whereas moderate-to-high heterogeneity (*I*^2^ ≥ 50%) necessitated the application of a random-effects model. Sensitivity analyses were run on studies with very high heterogeneity (>70%) and were conducted by sequentially excluding individual studies and reanalyzing the remaining data. Publication bias was assessed using Egger’s regression test for outcomes involving more than ten studies. A *P*-value < 0.05 was considered indicative of statistical significance.

## Results

The literature screening process is illustrated in Figure 1 (PRISMA flow diagram). A total of 3048 articles were identified across databases, including PubMed (910), the Cochrane Library (41), Embase (1187), and ScienceDirect (910). After removing duplicates, 2793 records remained. Following title and abstract screening, 20 studies were selected. Of these records, 4 conference abstracts were excluded, and 16 full-text articles were screened. Ultimately, 12 articles met the inclusion criteria and were incorporated into the final meta-analysis. The main characteristics of the included studies are summarized in Table 1, an overview of the key findings from the included studies is given in Table 2, and the quality assessment results are presented in Figures 2 and 3. The ICG protocols varied across studies, particularly in terms of dosage and dilution. Most studies administered ICG intravenously after ligation of the target segmental vessels and bronchus. The reported doses ranged from approximately 2 mL of diluted solution (5 mg/mL) to a total of 25 mg, with visualization of the intersegmental plane typically occurring within 10–20 seconds following injection, as shown in Table 3.

**Figure 1.**
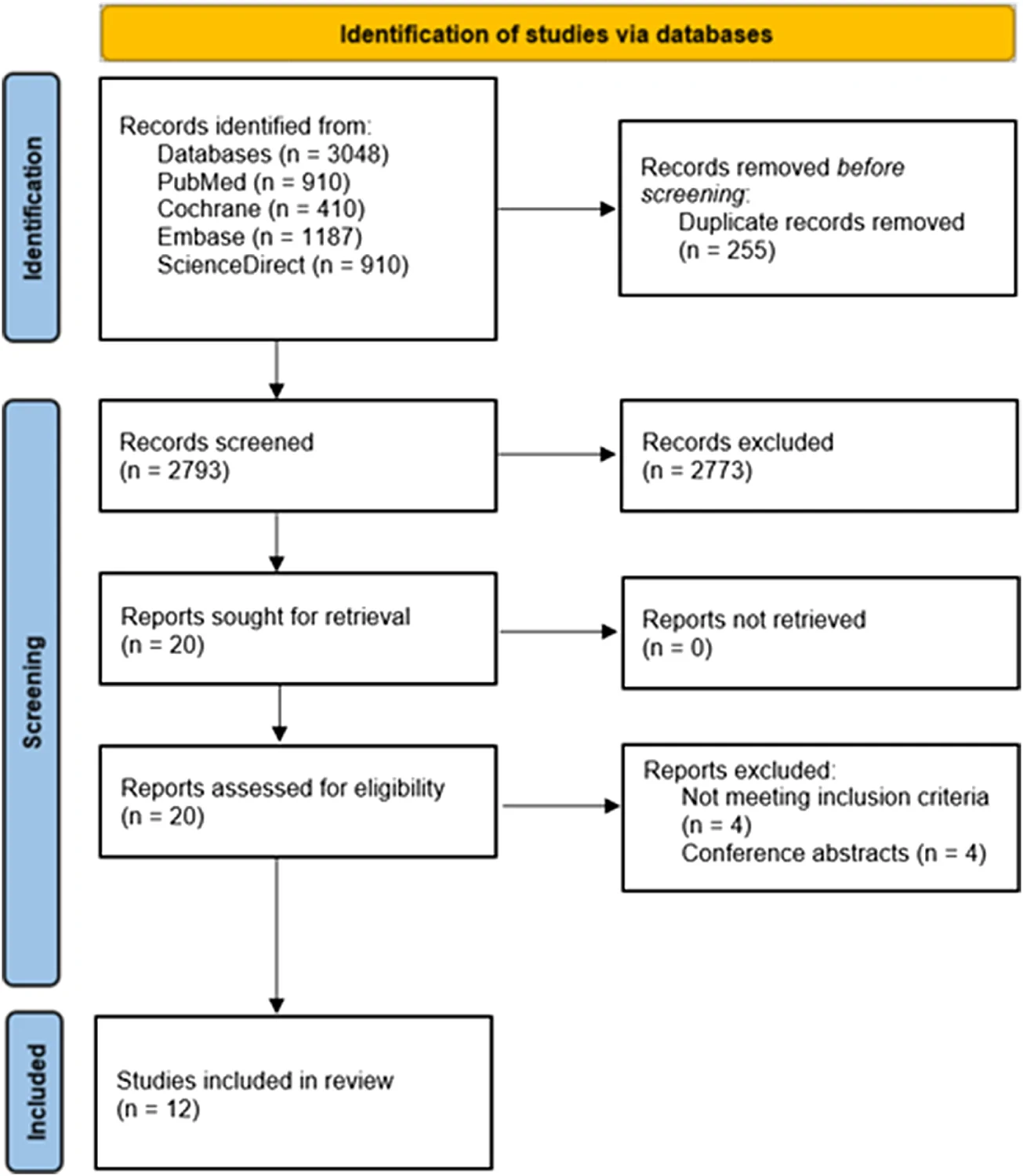
PRISMA Flow Diagram showing literature screening process.

**Figure 2.**
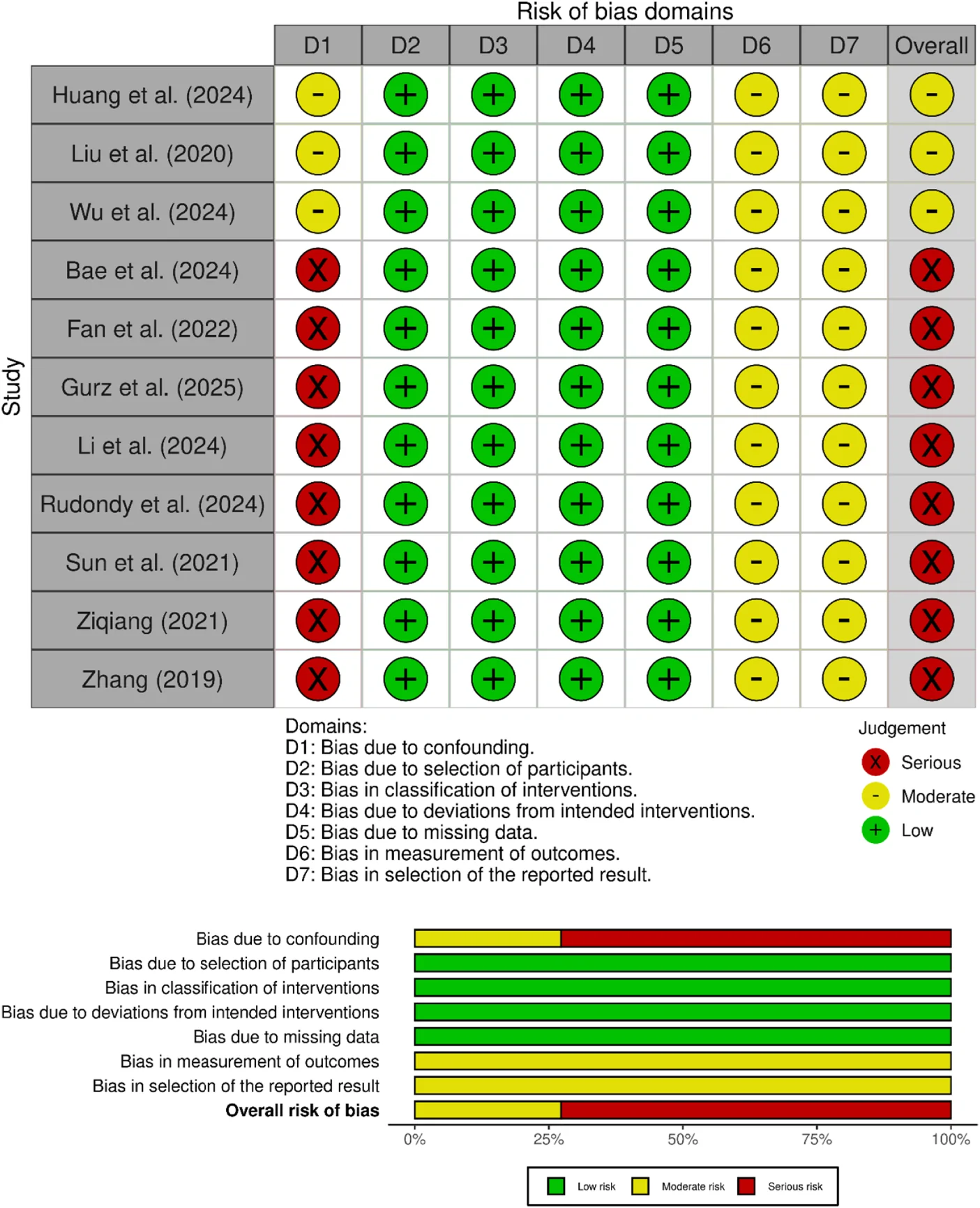
ROBINS-I assessment of the included non-randomized studies.

**Figure 3.**
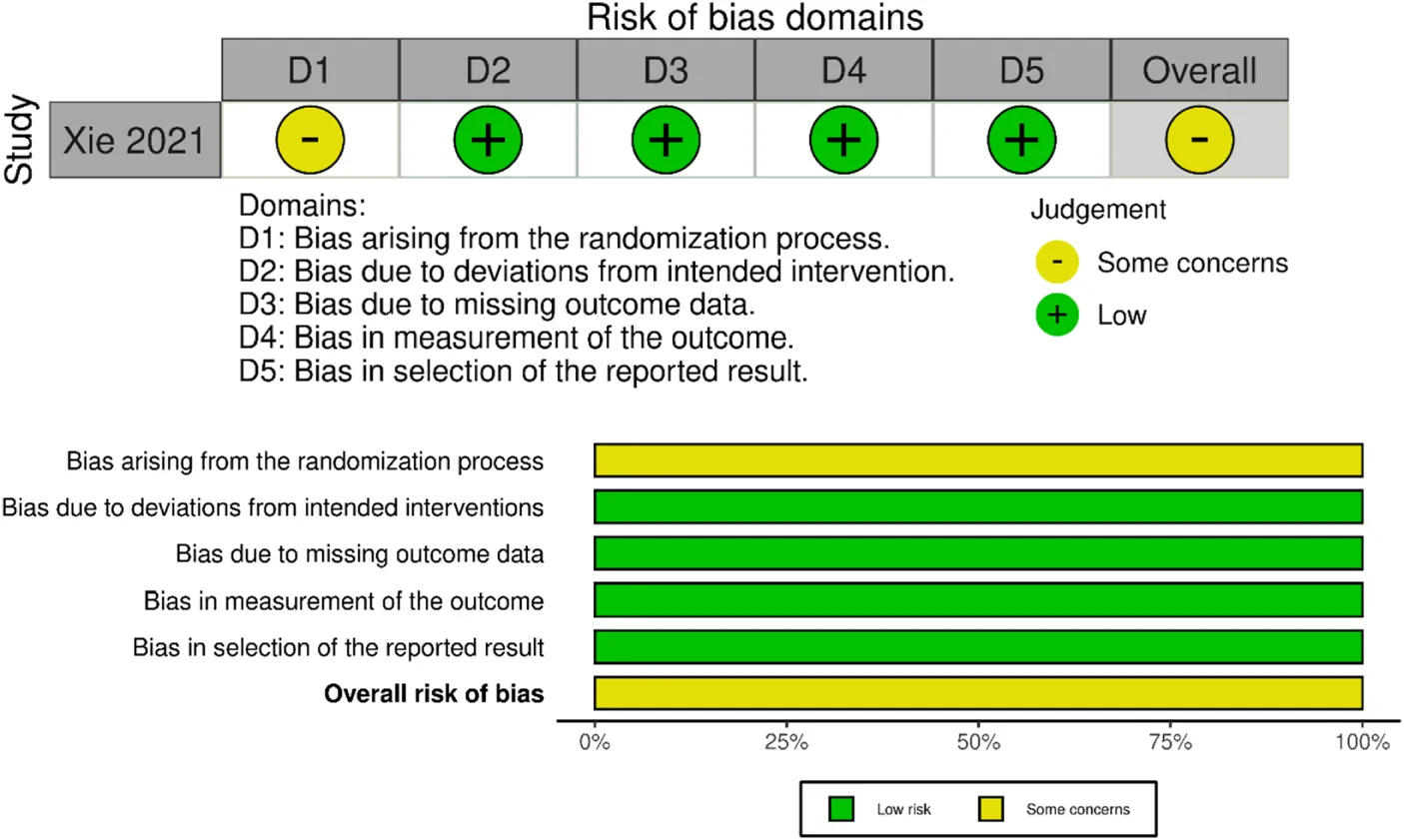
RoB 2.0 assessment of the included randomized studies.

**Table 1:** The baseline characteristics of included studies.

Author	Year	Sample size		Sex (M/F)		Median age		Surgery type
		ICG	Control	ICG	Control	ICG	Control	
Fan *et al*	2022	100	100	53/47	N/A	54	N/A	Uniport VATS segmentectomy
Gurz *et al*	2025	21	42	9/12	27/15	59.71	60.19	VATS anatomical segmentectomy
Huang *et al*	2024	66	66	18/48	16/50	53.58	53.32	VATS anatomical segmentectomy
Li *et al*	2024	50	51	20/30	20/31	57.1	54.5	Uniport VATS segmentectomy
Liu *et al*	2020	92	92	43/49	45/47	69.8	70.9	VATS anatomical segmentectomy
Rudondy *et al*	2024	124	73	49/75	31/42	66.6	67.5	Minimally invasive segmentectomy
Sun *et al*	2021	100	98	43/57	48/50	59.8	60.6	Uniport VATS anatomical segmentectomy
Wu *et al*	2024	65	65	24/41	27/38	52.06	53.8	VATS anatomical segmentectomy
Xie MR *et al*	2021	30	30	18/12	12/18	56.7	51.8	Uniport VATS anatomical segmentectomy
Bae *et al*	2024	58	261	32/26	125/136	65.1	64.7	VATS anatomical segmentectomy
Zhang	2019	60	97	41/62	19/35	55	56	VATS anatomical segmentectomy
Ziqiang	2021	26	40	10/13	16/27	56.65	56.08	Uniport VATS anatomical segmentectomy

VATS, video-assisted thoracoscopic surgery.

**Table 2:** Overview of the key findings from the included studies.

Outcomes	No. of studies	Effect model	MD/OR/RR	*I*^2 (%)^	Ph	Effect size (95% CI)	*P* value
Postoperative hemoptysis	5	Fixed	Risk Ratio (RR)	0%	0.51	0.89 (0.58, 1.37)	0.60
Atelectasis	3	Fixed	Risk Ratio (RR)	0%	0.88	1.48 (0.46, 4.74)	0.51
Pulmonary infection	6	Fixed	Risk Ratio (RR)	4%	0.39	0.89 (0.46, 1.73)	0.74
Postoperative air leak ≥1 day	4	Random	Risk Ratio (RR)	56%	0.08	0.42 (0.23, 1.02)	0.06
Prolonged air leak (>5 days)	2	Fixed	OR	0%	0.43	0.64 (0.31, 1.32)	0.23
Prolonged air leak (>3 days)	2	Fixed	OR	0%	0.70	0.93 (0.34, 2.53)	0.88
Pleural effusion	3	Fixed	OR	0%	0.92	0.78 (0.33, 1.82)	0.57
Unspecified complications	3	Fixed	OR	0%	0.64	0.71 (0.37, 1.38)	0.31
Operative time	10	Random	MD	96%	<0.00001	−21.09 (−33.24, −8.95)	0.0007
Intraoperative blood loss	8	Random	MD	81%	<0.00001	−12.29 (−21.21, −3.37)	0.007
Number of lymph nodes resected	7	Fixed	MD	0%	0.68	−0.05 (−0.17, 0.07)	0.40
Intersegmental plane visualization time	5	Random	MD	100%	<0.00001	−1212.61 (−1589.78, −835.44)	<0.00001
Postoperative length of hospitalization (days)	11	Random effect	MD	77 %	<0.00001	−0.47 (−0.84, −0.10)	0.01
Postoperative drainage time (days)	9	Random effect	MD	77 %	<0.00001	−0.41 (−0.94, 0.13)	0.14

**Table 3:** Summary of ICG administration protocols across included studies (dose, timing, and route).

Study	Dose	Timing	Route	Notes
Fan *et al*	5 mL ICG (concentration not reported)	After segmental dissection	IV	Used for intersegmental plane visualization
Gurz *et al*	Not reported	Intraoperative systemic injection	IV	Margin identification
Huang *et al*	2 mL bolus; 25 mg in 10 mL saline	During NIF imaging	IV	Visualization ~ 7–15 sec
Li *et al*	2 mL IV; total 25 mg	After vascular interruption	IV	Watershed analysis technique
Liu *et al*	6–10 mL (2.5 mg/mL); total 15–20 mg	Intraoperative	IV	Visualization ~15 sec
Rudondy *et al*	25 mg in 10 mL; ~5 mL injected	After segmental ligation	IV	Max 0.5 mg/kg
Sun *et al*	5 mg	After segmental dissection	IV	Low-dose protocol
Wu *et al*	Not reported	After segmental division	IV	Systemic injection described
Xie *et al*	25 mg in 25 mL; 3 mL injected	After dissection	IV	Visualization ~ 10–15 sec
Yong Bae *et al*	Not reported	Intraoperative use	IV	Not specified
Zhang *et al*	25 mg	After pulmonary artery division	IV	Reverse staining method
Ziqiang *et al*	Not reported	Intraoperative fluorescence	IV	Details not extractable

ICG, indocyanine green; IV, intravenous; NIF, near-infrared fluorescence imaging.

### Intraoperative outcomes

The studies that reported operative time demonstrated that ICG guidance significantly reduced operative time compared with conventional methods (MD = −21.09 minutes, 95% CI: −33.24 to −8.95, *P* = 0.0007) (Fig. 4). Heterogeneity was high (*I*^2^ = 96%), which likely reflects inconsistencies in the measurement of operative time across institutions, particularly differences in the starting point of recording. Sensitivity analyses demonstrated that removing individual studies, including those with the largest effect sizes, did not materially change the direction of effect, and all leave-one-out analyses continued to favor ICG. Although the magnitude of the pooled effect decreased slightly after excluding outlier studies, the overall benefit remained, indicating that the finding is robust despite heterogeneity. Funnel plot assessment and Egger’s regression test (*t* = −0.80, *P* = 0.45) showed no significant publication bias.

**Figure 4.**
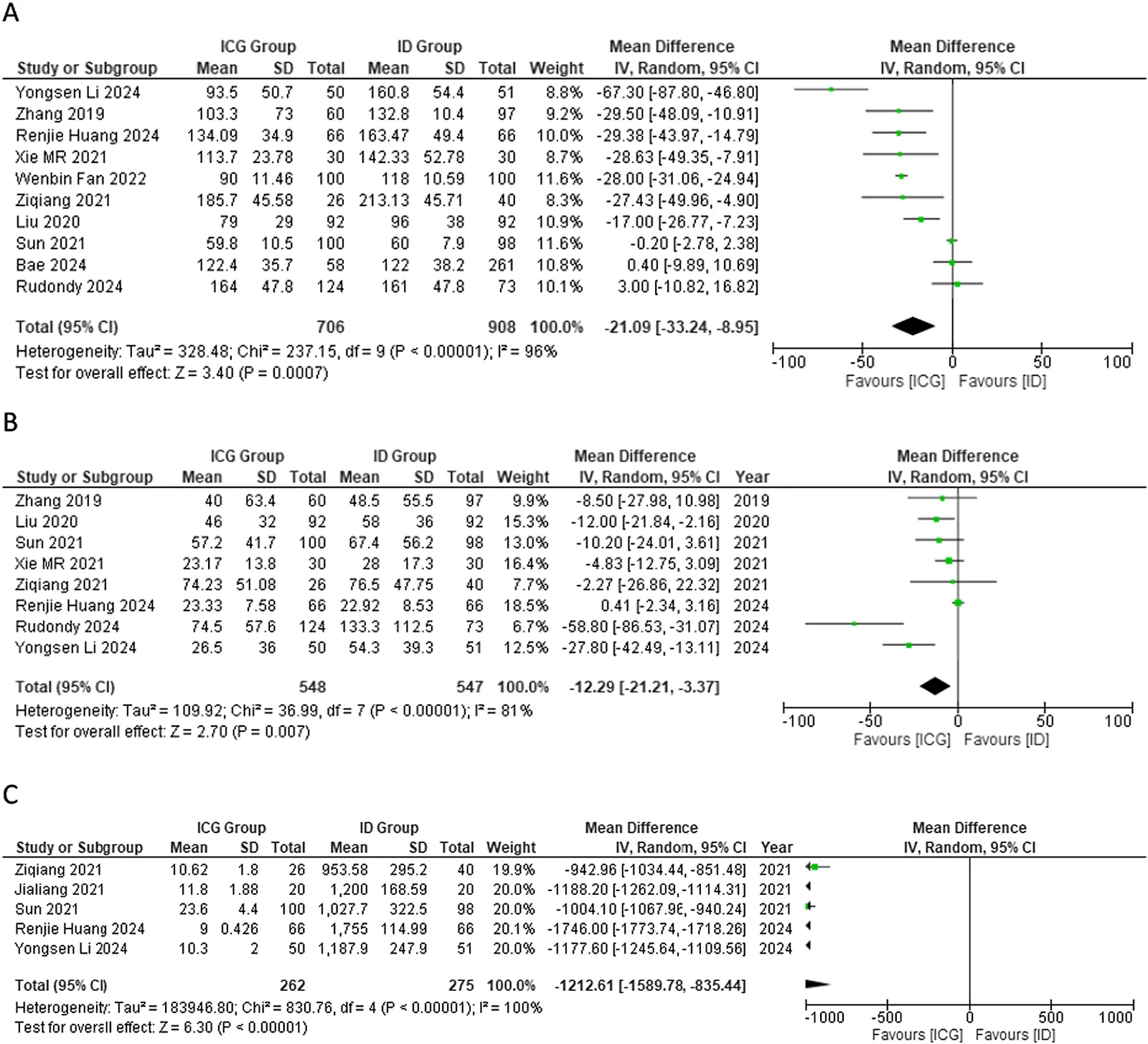
Forest plots comparing ICG-FI and IDM. (A) Operative time (min). (B). Intraoperative blood loss (mL). (C). Intersegmental plane visualization time (seconds).

Furthermore, the pooled analysis also revealed significantly lower blood loss in the ICG group (MD = −12.29 mL, 95% CI: −21.21 to −3.37, *P* = 0.007, *I*^2^ = 81%) (Fig. 4). While the absolute difference was small, the consistent effect across studies suggests improved visualization and hemostasis with ICG fluorescence guidance. ICG also significantly shortened intersegmental plane visualization time (MD = −20.21 minutes; 95% CI: −26.49 to −13.92, *P* < 0.00001) (Fig. 4). Despite high heterogeneity (*I*^2^ = 100%), all included studies demonstrated a substantial and consistent advantage for ICG in delineating intersegmental planes. There was no significant difference in the number of lymph nodes resected between the ICG and control groups (MD = −0.05, 95% CI: −0.17 to 0.07, *P* = 0.40, *I*^2^ = 0%), indicating that ICG use does not compromise oncological thoroughness. A detailed overview of the pooled effect sizes and key findings for all outcomes is provided in Table 4.

**Table 4:** Study design and characteristics of included studies.

Study	Study Design	Population/Comparison
Fan *et al*	Retrospective comparative study	ICGF vs inflation–deflation
Gurz *et al*	Retrospective comparative study	ICG vs inflation–deflation
Huang *et al*	Single-center retrospective (PSM)	3DP + ICG vs CTBA-ID
Li *et al*	Retrospective study	Watershed + ICG vs MID
Liu *et al*	Retrospective PSM study	NIF-VATS vs ID-VATS
Rudondy *et al*	Retrospective database analysis	ICG vs MID
Sun *et al*	Retrospective comparative study	Uniport VATS (ICG vs MID)
Wu *et al*	Multicenter retrospective (PSM)	Fluorescence vs inflation–deflation
Xie MR *et al*	Prospective randomized controlled trial	Precision fluorescence vs traditional
Bae *et al*	Single-center retrospective study	ICG vs inflation–deflation
Zhang *et al*	Retrospective case-control study	Fluorescence vs MID
Ziqiang *et al*	Retrospective study (Master’s thesis)	ICG fluorescence application

ICG, indocyanine green; VATS, video-assisted thoracoscopic surgery; MID, modified inflation–deflation method; NIF, near-infrared fluorescence; PSM, propensity score matching; CTBA, computed tomography bronchography and angiography; 3DP, three-dimensional printing.

### Postoperative outcomes

Two studies assessed air leaks lasting more than 5 days. The pooled analysis of these studies found no significant difference between the ICG and control groups (OR = 0.64, 95% CI: 0.31–1.32, *P* = 0.23, *I*^2^ = 0%). Air leaks lasting more than 3 days were reported in two studies. No significant difference was observed in the analysis (OR = 0.93, 95% CI: 0.34–2.53, *P* = 0.88, *I*^2^ = 0%). Initial analysis of studies that reported postoperative air leaks for at least 1 day showed a non-significant trend toward fewer air leaks in the ICG group (OR = 0.42, 95% CI: 0.16–1.06, *P* = 0.07, *I*^2^ = 63%) (Fig. 5). After excluding one study (Zhang 2019) as a potential outlier, the sensitivity analysis revealed a statistically significant reduction (RR = 0.26, 95% CI: 0.21–0.59, *P* < 0.0001, *I*^2^ = 0%), confirming a meaningful decrease in postoperative air leaks with ICG use (Fig. 5).

**Figure 5.**
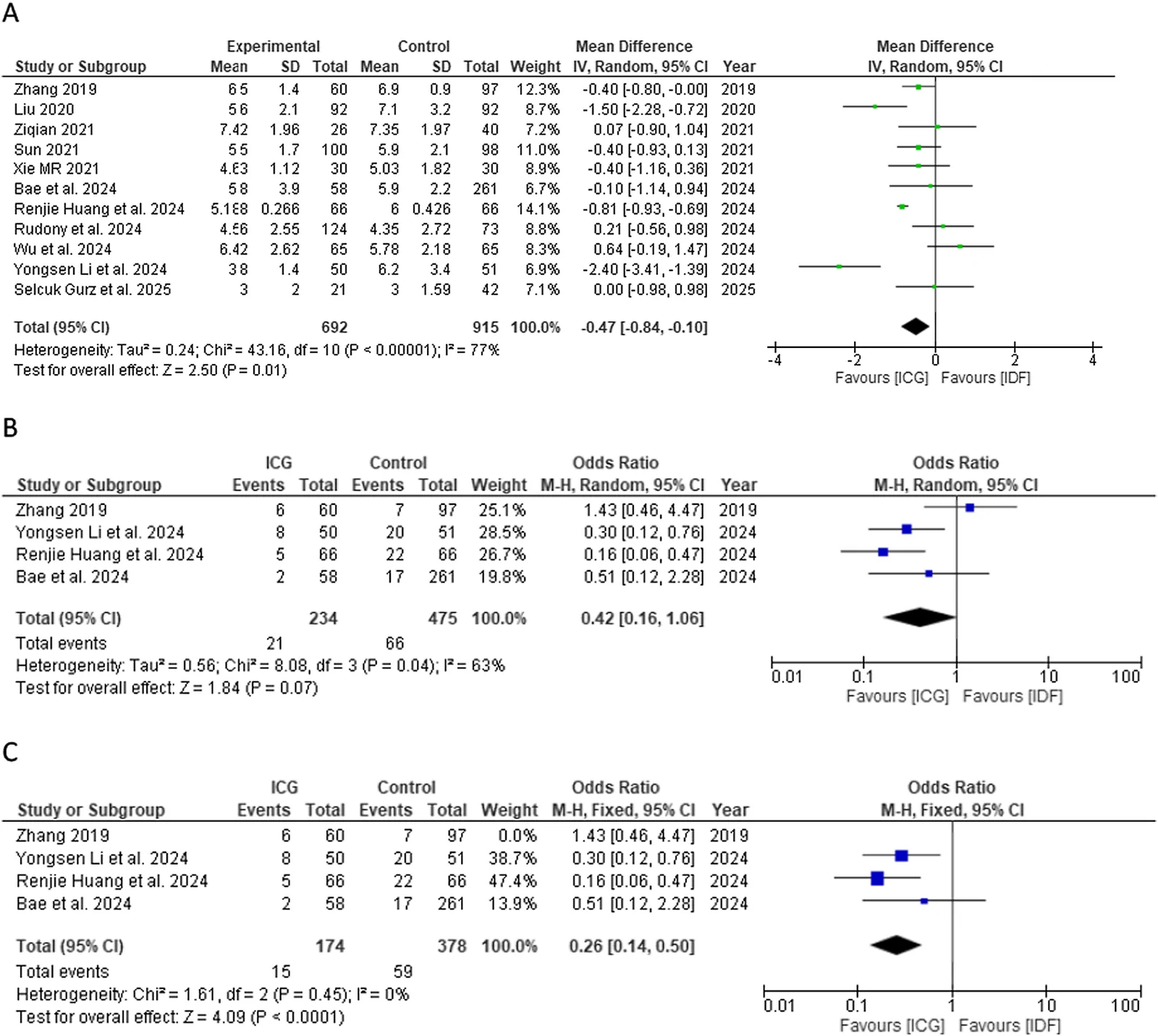
Forest plots of postoperative outcomes. (A) Post-operative hospitalization time. (B) Post-operative air leakage (pre-sensitivity analysis). (C) Post-operative air leakage (post-sensitivity analysis).

Among the three studies that reported postoperative pleural edema, no significant difference was observed (OR = 0.78, 95% CI: 0.33–1.82, *P* = 0.57, *I*^2^ = 0%), though all studies trended in favor of ICG. Postoperative pulmonary infection was evaluated in six studies. The pooled results showed no significant difference between ICG and control groups (OR = 0.89, 95% CI: 0.44–1.79, *P* = 0.74, *I*^2^ = 6%), suggesting consistent and comparable infection rates. Five studies reported hemoptysis and three reported atelectasis. No significant differences were found for either outcome (hemoptysis: OR = 0.87, 95% CI: 0.51–1.47, *P* = 0.51; atelectasis: OR = 1.50, 95% CI: 0.45–4.94, *P* = 0.88; *I*^2^ = 0%), indicating similar safety profiles. Unspecified postoperative complications were reported in three studies. No significant difference was observed (OR = 0.71, 95% CI: 0.37–1.38, *P* = 0.31, *I*^2^ = 0%), confirming the overall safety of the ICG-guided procedure.

The pooled analysis demonstrated no statistically significant difference in postoperative drainage time between the ICG-guided (experimental) and control groups (MD = −0.41; 95% CI: −0.94 to 0.13; *P* = 0.14; *I*^2^ = 77%). Although the MDs across studies tended to favor the experimental group (indicating lower postoperative drainage time), the overall effect was not statistically significant (*P* ≥ 0.05). The high level of heterogeneity suggests interstudy variability, potentially due to differences in patient selection, perioperative management, or surgical techniques. The meta-analysis revealed that ICG-guided procedures were associated with a statistically significant reduction in postoperative hospitalization time compared with the control group (MD = −0.47; 95% CI: −0.84 to −0.10; *P* = 0.01; *I*^2^ = 77%) (Fig. 5). Despite the heterogeneity, the direction of effect across studies indicates that the experimental intervention significantly reduced the length of hospital stay (*P* < 0.05). Assessment of publication bias using Egger’s regression test showed no statistically significant funnel plot asymmetry (*t* = 1.39, *P* = 0.199), indicating that the findings were unlikely to be influenced by small-study effects.

### Sensitivity analysis

Sensitivity analyses were performed for all primary postoperative outcomes, including postoperative hospital stay, drainage time, operative time, and intraoperative blood loss. The leave-one-out analyses confirmed that the overall direction of effect remained consistent across all outcomes, thereby affirming the validity of the findings.

For postoperative length of hospital stay, the exclusion of Wu *et al* (2024) did not significantly change the effect estimate (MD: −0.47 to −0.57), with heterogeneity decreasing slightly from 77 to 72%. Similarly, postoperative drainage time remained non-significant after removing Yongsen *et al* (2014), and heterogeneity decreased from 77 to 52%, suggesting that variability was partly driven by this study.

For operative time, exclusion of Sun *et al* (2021) resulted in a minor change in magnitude (MD: −21.09 to −23.45 minutes) and a reduction in heterogeneity from 96 to 87%, confirming that ICG consistently shortened operative time across studies. A similar pattern was observed for intraoperative blood loss, where exclusion of Huang *et al* (2024) increased the effect size modestly (MD: −12.29 to −15.16 mL) and reduced heterogeneity from 81 to 69%. Across all analyses, the direction of effect remained unchanged, indicating that no single study disproportionately influenced the pooled results.

### Risk of bias assessment

Risk of bias was evaluated using ROBINS-I for observational studies and the RoB 2.0 tool for the single randomized controlled trial. Most observational studies demonstrated a moderate risk of bias in domains related to confounding, outcome measurement, and reporting, while several studies were judged to have a serious risk of bias, primarily due to uncontrolled confounding and participant selection. Despite these concerns, all studies showed a low risk of bias in domains related to the classification of interventions and missing data.

The randomized controlled trial (Xie 2021) included in the analysis demonstrated an overall moderate risk of bias, with concerns primarily related to the randomization process. No domains were rated as high risk. Overall, the included studies were considered acceptable for quantitative synthesis, but the predominance of moderate-to-serious risk of bias highlights the need for cautious interpretation of the pooled findings.

## Discussion

This meta-analysis evaluated the impact of ICG-FI on peri- and postoperative outcomes in thoracoscopic segmentectomy. Across the 11 included studies, ICG-FI was consistently associated with shorter operative time, lower intraoperative blood loss, and faster visualization of the intersegmental plane. Although most postoperative complications showed no significant difference between the ICG and control groups, a sensitivity analysis revealed that fluorescence guidance significantly reduced postoperative air leaks when an outlier study was excluded. Together, these findings highlight the potential of ICG-FI to enhance surgical precision, efficiency, and recovery while maintaining oncologic completeness. Clinically, ICG-FI is especially useful in patients with poor lung function or complex anatomy, where traditional methods may fail to clearly define the intersegmental plane. Several outcomes demonstrated substantial heterogeneity, particularly operative time (*I*^2^ = 96%) and intersegmental plane visualization time (*I*^2^ = 100%). This heterogeneity likely reflects variations in clinical and methodological factors across studies, including differences in ICG dosage, timing of injection, route of administration, surgical expertise, and patient characteristics. Furthermore, variations in study design and perioperative management protocols may have contributed to the observed variability. Due to limited reporting and the relatively small number of studies per outcome, subgroup analyses or meta-regression were not feasible. Therefore, these findings should be interpreted with caution.

Despite advances in diagnosis and treatment, lung cancer remains the leading cause of cancer-related mortality worldwide, accounting for an estimated 1.8 million deaths annually^[^9^]^. A major contributor to this burden is the high proportion of cases detected at advanced stages, when curative resection is no longer feasible. The increasing adoption of low-dose computed tomography screening has changed the approach by enabling the detection of small, localized pulmonary nodules amenable to surgical intervention^[^10^]^. As a result, anatomical segmentectomy has gained prominence as a lung-sparing alternative to lobectomy, offering comparable oncologic efficacy while preserving pulmonary function^[^11^]^. Nevertheless, successful segmentectomy depends on the surgeon’s ability to identify the intersegmental plane precisely – an area where ICG-FI has shown promise in improving anatomical visualization and operative efficiency.

ICG binds to plasma proteins and emits near-infrared fluorescence when excited by infrared light, enabling surgeons to visualize the perfusion status of pulmonary segments in real time^[^12^]^. ICG-FI has been widely adopted in other surgical specialties, such as hepatobiliary, colorectal, and breast surgery, for vascular and lymphatic mapping and has now become an invaluable tool in thoracic procedures^[^13,14^]^. By intravenously injecting ICG after the target segmental artery is divided, the fluorescent boundary accurately represents the perfused (fluorescent) and non-perfused (non-fluorescent) regions, allowing precise demarcation of the intersegmental plane without the need for inflation-deflation maneuvers^[^15^]^.

Traditional methods for delineating intersegmental borders rely on either ventilation differences (modified inflation–deflation, selective bronchial ventilation) or circulatory differences (temporary vessel occlusion or contrast injection)^[^16^]^. Ventilation-based approaches are often unreliable due to collateral ventilation through the pores of Kohn and Lambert channels, leading to blurring of segmental margins^[^17,18^]^. Circulation-based techniques are more accurate but technically demanding, as they require temporary pulmonary artery ligation or selective dye injection, extending operative time and complexity^[^19,20^]^. In contrast, ICG-FI provides a rapid, reproducible, and minimally invasive alternative that improves visualization with minimal physiological interference.

The pooled results of this meta-analysis are consistent with previous reports showing that fluorescence-guided navigation enhances efficiency and safety in thoracoscopic segmentectomy by enabling surgeons to rapidly and objectively define the intersegmental plane^[^8^]^.

The improved outcomes with ICG-FI can be linked to several mechanisms. First, real-time fluorescence imaging provides objective perfusion mapping, allowing surgeons to make accurate cuts along true anatomical borders and minimize unnecessary trauma^[^21^]^. Second, the method eliminates the waiting time for lung inflation-deflation and prevents incomplete re-expansion, thus shortening operative time and improving field visibility^[^22^]^. Third, precise identification of segmental borders may reduce the risk of prolonged air leaks, which are common after thoracoscopic segmentectomy due to air leakage from misidentified parenchymal margins^[^17^]^. In this analysis, the ICG-FI group demonstrated a trend toward fewer postoperative complications, consistent with the hypothesis that improved anatomic precision translates into faster recovery.

Despite the strengths of this meta-analysis, several limitations should be acknowledged. There was considerable heterogeneity among the included studies, particularly regarding surgical techniques, patient characteristics, and fluorescence protocols, which may have influenced the pooled estimates. Although the overall sample size was substantial, most of the included studies were retrospective cohorts and had a moderate to serious risk of bias, especially due to confounding and patient selection. This may have led to overestimation of the benefits of ICG-FI, as patients in the ICG group may have had more favorable characteristics or may have been treated in more experienced centers. Therefore, the results should be interpreted with caution and cannot confirm causality. In addition, the possibility of publication bias and bias related to non-randomized study designs cannot be excluded. The use of heterogeneous endpoints and varied definitions of postoperative outcomes across studies may also have contributed to inconsistency.

Moreover, the present results should not be interpreted as proof of the universal superiority of ICG-FI. Although ICG-FI typically provides clear segmental borders, its performance may be reduced in emphysematous lungs or in segments with impaired perfusion, where fluorescence contrast is insufficient for optimal visualization. Furthermore, widespread implementation is affected by a non-negligible learning curve and additional equipment-related costs, which may limit availability in resource-constrained centers.

The high heterogeneity observed across studies likely reflects differences in ICG protocols, including dose, timing of administration, and surgical technique, highlighting the lack of standardized approaches in current practice. From a clinical standpoint, this variability may limit reproducibility and consistent application of ICG-FI across institutions. Therefore, there is a need to establish standardized fluorescence protocols to optimize its use. In the future, the integration of artificial intelligence and advanced health informatics could play a vital role in overcoming these standardization barriers. For instance, large language models like ChatGPT are increasingly being evaluated for their utility across diverse healthcare scenarios, including clinical decision support, medical education, and literature synthesis^[^23^]^. In the context of thoracoscopic segmentectomy, leveraging such global-scale machine learning tools could eventually assist clinicians in synthesizing complex, patient-specific data to recommend optimal ICG-FI parameters such as dye dosage and timing, thereby mitigating the learning curve and improving institutional reproducibility. In addition, future large-scale, multicenter randomized controlled trials are required to validate these findings and assess long-term oncologic and functional outcomes. Specifically, there is a need for standardized fluorescence imaging protocols that include dye dosage, timing of administration, and camera settings to improve reproducibility across institutions. High-quality multicenter prospective randomized controlled trials with long-term oncologic and functional follow-up are also required to further validate the clinical value of ICG-FI and refine patient selection.

## Conclusion

This systematic review and meta-analysis demonstrate that ICG-FI is a better alternative to the modified IDM for identifying intersegmental planes during thoracoscopic segmentectomy. The pooled data show that ICG-FI significantly improves intraoperative efficiency by reducing operative time, minimizing blood loss, and providing rapid, real-time visualization of intersegmental boundaries. Moreover, these improvements are achieved without compromising oncological adequacy, as both groups had comparable lymph node yields.

The ICG-FI group also showed a significant reduction in the length of hospital stay. Although both techniques showed similar complication rates, a sensitivity analysis indicates that fluorescence guidance effectively reduces the incidence of postoperative air leaks, likely due to more precise parenchymal demarcation. While there was significant heterogeneity, the benefits of ICG-FI were consistent. Future research should focus on large-scale randomized trials to standardize fluorescence protocols and validate long-term functional and oncologic safety.

## Data Availability

The corresponding authors confirm that the data supporting the findings of this study are available within the article. Raw data that support the findings of this study are available from the corresponding authors upon request.

## Research registration unique identifying number (UIN)

CRD420251133611.
